# Lung Ultrasonography Accuracy for Diagnosis of Adult Pneumonia: Systematic Review and Meta-Analysis

**DOI:** 10.3390/arm92030024

**Published:** 2024-06-04

**Authors:** Dev Desai, Abhijay B. Shah, Joseph Rem C. Dela, Tayba A. Mugibel, Khalid M. Sumaily, Essa M. Sabi, Ahmed H. Mujamammi, Maria E. Malafi, Sara A. Alkaff, Thurya A. Alwahbi, Jamal O. Bahabara, Lotfi S. Bin Dahman

**Affiliations:** 1 Nathiba Hargovandas Lakhmichand (NHL) Municipal Medical College, Gujarat University, Ahmedabad 380006, India; devhedsai01@gmail.com (D.D.); abhijayshah0610@gmail.com (A.B.S.); 2 College of Medicine, University of the Philippines, Manila 1000, Philippines; jcdelacruz13@up.edu.ph; 3 College of Medicine and Health Sciences, Hadhramout University, Mukalla, Yemen; saraalkaff99@gmail.com (S.A.A.); thuryaalwahbi@gmail.com (T.A.A.); 4 Clinical Biochemistry Unit, Laboratory Medicine Department, College of Medicine and Health Sciences, Hadhramout University, Mukalla, Yemen; lotfydahman@hu.edu.ye; 5 Clinical Biochemistry Unit, Pathology Department, College of Medicine, King Saud University, Riyadh 11461, Saudi Arabia; ksumaily@ksu.edu.sa (K.M.S.); esabi@ksu.edu.sa (E.M.S.); amujamammi@ksu.edu.sa (A.H.M.); 6 Medical School, Democritus University, 68100 Alexandroupolis, Greece; marilenmalafi@gmail.com; 7 Radiology Unit, Department of Specialized Surgery, College of Medicine and Health Sciences, Hadhramout University, Mukalla, Yemen; bahabroo64@gmail.com; 8 Hadhramout Foundation—Human Development, Mukalla, Yemen

**Keywords:** pneumonia, lung ultrasound, meta-analysis

## Abstract

**Highlights:**

**What are the main findings?**

1. Ultrasonography (USG) can be used to diagnose adult pneumonia as it has excellent diagnostic performance including sensitivity and specificity.
2. Bedside USG can be done in patients where pneumonia is suspected.

**What is the implication of the main finding?**

3. This ensures the correct and easier way of diagnosis in regions where X-ray machines are not used, such as in remote and poor areas or even in patients home.
4. The portability and the easier learning arc for non-radiologist doctors is a huge benefit in the world of diagnostics.

**Abstract:**

Background: Pneumonia is a ubiquitous health condition with severe outcomes. The advancement of ultrasonography techniques allows its application in evaluating pulmonary diseases, providing safer and accessible bedside therapeutic decisions compared to chest X-ray and chest computed tomography (CT) scan. Because of its aforementioned benefits, we aimed to confirm the diagnostic accuracy of lung ultrasound (LUS) for pneumonia in adults. Methods: A systematic literature search was performed of Medline, Cochrane and Crossref, independently by two authors. The selection of studies proceeded based on specific inclusion and exclusion criteria without restrictions to particular study designs, language or publication dates and was followed by data extraction. The gold standard reference in the included studies was chest X-ray/CT scan or both. Results: Twenty-nine (29) studies containing 6702 participants were included in our meta-analysis. Pooled sensitivity, specificity and PPV were 92% (95% CI: 91–93%), 94% (95% CI: 94 to 95%) and 93% (95% CI: 89 to 96%), respectively. Pooled positive and negative likelihood ratios were 16 (95% CI: 14 to 19) and 0.08 (95% CI: 0.07 to 0.09). The area under the ROC curve of LUS was 0. 9712. Conclusions: LUS has high diagnostic accuracy in adult pneumonia. Its contribution could form an optimistic clue in future updates considering this condition.

## 1. Introduction

Pneumonia is a significant healthcare and economic issue with a massive impact on morbidity and mortality, ranking as the third leading cause of death globally [1,2,3,4,5,6]. It is a primary infectious killer and one of the most frequent causes of ER visits and hospital admissions [7,8]. In addition, it is the second-most-prevalent nosocomial infection with the greatest fatality rate, making it not only a reason for hospital admission, but also a significant healthcare-related complication [9].

In developed countries, the overall prevalence of community-acquired pneumonia (CAP) varies from 1.6 to 16 cases per 1000 and about 20% of them require hospitalization, with a fatality rate as high as 48% [10]. Owing to this heavy burden, it is a continuous struggle for doctors to differentiate pneumonia from other differential diagnoses through clinical presentation alone in order to start effective treatment—especially in the backdrop of antimicrobial resistance [11,12].

A combination of suggestive clinical signs and the presence of consolidation or opacification on a chest x-ray (CXR) or computerized tomography (CT) scan of the chest is used to make the diagnosis of pneumonia [13,14]. Currently, the most common initial approach in cases of possible pneumonia is chest X-ray (CXR), especially in low–middle income countries (LMICs) [15,16,17]. However, it has many restrictions, such as that it cannot be used on pregnant women due to radiation exposure, and its requirement of both poster anterior and lateral projections in hospitalized patients, particularly in the critically ill [18,19]. Meanwhile, the gold-standard imaging method for pneumonia, the chest CT scan, has its own disadvantages, such as being more expensive, impractical and exposing patients to more radiation than CXR [19,20,21]. Both techniques are time-consuming, and radiologists have many disagreements on the interpretation of the results [22,23].

Although being previously restricted to the identification of pleural effusions, thoracentesis and biopsy-guided treatments, ultrasonography techniques have significantly advanced in recent years in evaluating pulmonary diseases such as pneumonia and pneumothorax [24,25,26,27,28]. In the last decade, LUS has grown in popularity in intensive care units and emergency departments, and has gained more acceptance as a potentially helpful diagnostic technique for community-acquired pneumonia [29,30], because it permits therapeutic decisions to be made at the bedside, is simple to repeat and prevents the patient from being exposed to ionizing radiation [26,27,28]. Thus, this study is focused on confirming the diagnostic accuracy of the LUS in diagnosing pneumonia through a systematic review and a meta-analysis that assembles several studies published in the literature.

## 2. Materials and Methods

### 2.1. Search Strategy and Study Eligibility

A systematic literature search was applied to Medline, Cochrane and Crossref. The terms “ultrasound”, “ultrasonography”, “sonograph”, and “pneumonia” were used in various combinations for carrying out the literature search. Only published researches were considered without any language restriction. The search of studies was not limited on the basis of publication dates or study designs. All prospective, retrospective and cross-sectional studies were included if meeting the following criteria: (1) adult patients aged ≥ 18 years with either clinical suspicion or confirmed diagnosis of pneumonia or acute respiratory failure; (2) enrollment of patients with community-acquired or nosocomial pneumonia including VAP; (3) reference method for diagnosing pneumonia was based on clinical data, laboratory results and confirmation by chest radiology/CT scan or both; (4) ability to extract the necessary data for calculating sensitivity and specificity. We excluded studies that enrolled children [31,32], included fewer than 20 participants [33] and studies that evaluated pneumonia only based on clinical data. The literature search and data analysis were carried out in February 2023.

### 2.2. Selection of Studies and Data Extraction

Two authors independently performed the search of the literature and screened the title and abstract of each article. Full-text articles that met the inclusion criteria were retrieved for this review. Any discrepancies during the entire process were resolved by consensus. The following data were extracted from each study: first author’s name, year of publication, country of origin, study design and setting, sample size, mean age and sex of the population, inclusion criteria expertise of operator, ultrasound diagnostic criteria considered in each study, and reference diagnostic standard.

### 2.3. Quality Assessment

The methodological quality was assessed using the Quality Assessment of Diagnostic Accuracy Studies (QUADAS-2) criteria [34], which provides a standardized approach for grading the quality of studies included in meta-analyses of diagnostic accuracy. The risk of bias and study generalizability are categorized by QUADAS-2 as low, unclear or high. Two authors scored the QUADAS-2 checklist independently and any disagreements were resolved via consensus.

### 2.4. Statistical Analysis

All statistical analyses were carried out using RevMan (Review Manager, version 5.3), SPSS (Statistical Package for the Social Sciences, version 20) and Excel in Stata 14. Individual study sensitivity and specificity were plotted on a Forest plot and the overall area under the receiver operating characteristic (ROC) curve was calculated. The post-test probabilities were calculated using the prior probability, and the summary positive and negative likelihood ratios, evaluated using the Fagan plot analysis command in Stata 14.0.

## 3. Results

### 3.1. Flowchart of Articles Retrieved from Search of Databases

**Figure 1:**
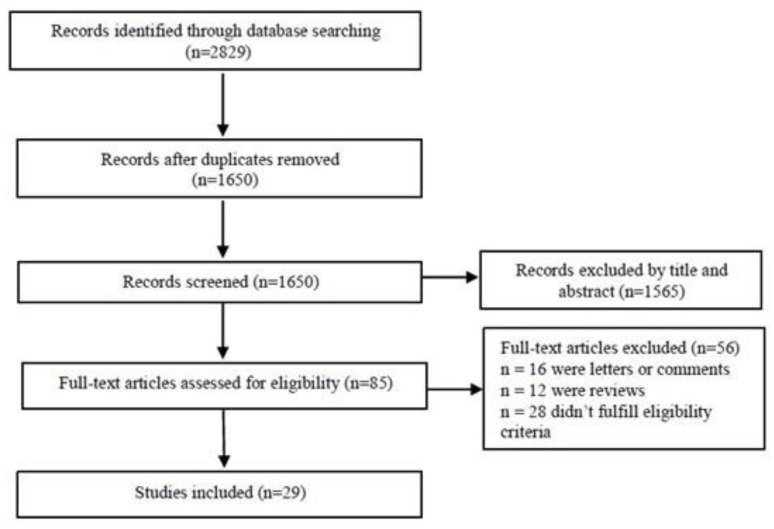
Flowchart of articles retrieved from search of databases.

A systematic search to retrieve studies that assessed the diagnostic accuracy of LUS for pneumonia in adults was performed in the Medline, Cochrane and Crossref databases. A total of 2829 studies were identified. After the first screening stage (title, abstract and keywords), 85 relevant studies were retrieved, and their full texts were reviewed for eligibility. A total of 29 studies with 6702 participants satisfying the inclusion criteria were analyzed (Figure 1). The study characteristics are shown in Table 1.

### 3.2. Main Characteristics of the Included Studies

In total, 13 (44.8%) studies were carried out in Italy, 5 (17.2%) in France and the remaining studies were carried out in USA, Iran, Egypt, Germany, Denmark, Switzerland, China and Turkey. The predominant design was prospective, three studies were cross-sectional [16,46,47] and only one study was performed retrospectively [54]. Final diagnosis considering all the observed instrumental and laboratory findings was the reference standard in ten (34.5%) studies [1,7,8,38,40,45,47,49,55,56]. Three studies (10.3%) used a combination of clinical criteria and imaging [35,41,52] and 16 (55.2%) used imaging only as the reference standard; seven used chest CT scan for the diagnosis of pneumonia in the entire sample and eight used chest CT scan when the results of CXR and LUS were found to be discordant. In one study, the reference standard was only the CXR [54]. Most studies reported blinding the professionals performing ultrasound to the results of the reference standard. Only five reported the absence of blinding, and three did not clearly state whether blinding took place.

A total of 20 studies were conducted in adult patients admitted to EDs and/or medical wards, 8 studies included critically ill patients in the ICU and one in the stroke unit [16]. The participant characteristics are shown in Table 2.

### 3.3. Forest Plots of Sensitivity and Specificity for Diagnosis of Pneumonia in Adults

**Figure 2:**
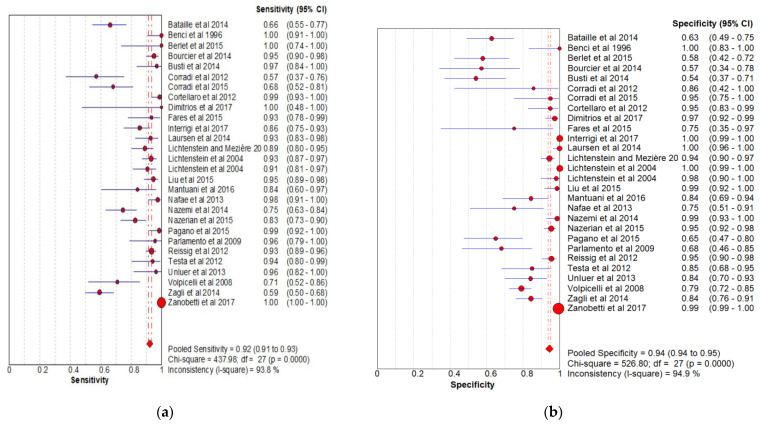
(**a**) Forest plot for sensitivity; (**b**) forest plot for specificity (LUS has an overall sensitivity of 92% (95% CI, 91–93%) and specificity of 94% (95% CI, 94–95%) in the diagnosis of pneumonia in adult).

The sensitivity and specificity of the considered studies are shown in the forest plot (Figure 2). Overall pooled sensitivity, specificity and PPV were 92% (95% CI, 91 to 93%), 94% (95% CI, 94 to 95%) and 93% (95% CI, 89 to 96%), respectively.

### 3.4. Positive and Negative Likelihood Ratio Using Fagan Plot Analysis

**Figure 3:**
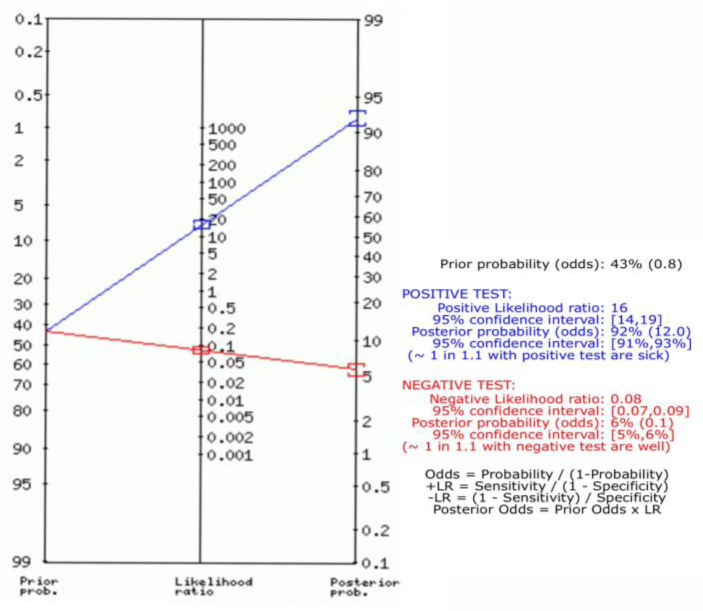
Fagan plot analysis showed the prior probability is 43, the positive likelihood ratio is 16, the probability of post-test is 92, the negative likelihood ratio is 0.08, and the probability of the post-test is 6.

Pooled positive and negative likelihood ratios were 16 (95% CI, 14 to 19) and 0.08 (95% CI, 0.07 to 0.09), respectively (Figure 3).

### 3.5. Receiver Operating Characteristic Curve for LUS in All Studies

**Figure 4:**
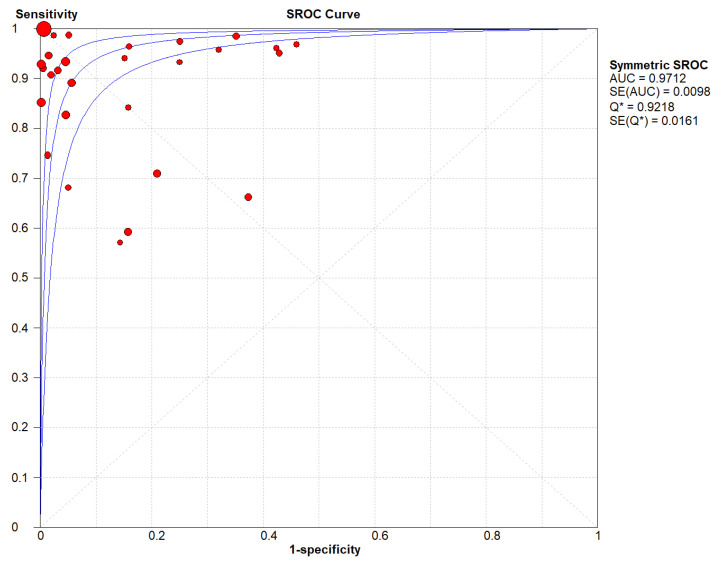
Summary receiver operating characteristic curve for LUS in all studies. The area under the ROC was 0.9712. The overall diagnostic odds ratio as per random effect model was 139.65 (57.02–342.02). The red dot in the SROC plot is each individual paper with the size of the ball corresponding to the same sample of the paper and in turn the weight of the paper in the analysis. The blue lines demonstrate the SROC curve approximations.

The estimation of the area under the ROC curve of lung ultrasound for the diagnosis of pneumonia was 0.9712. The overall diagnostic odds ratio as per random effect model was 139.65 (57.02–342.02) (Figure 4).

### 3.6. Risk of Bias and Applicability Concerns of Included Studies

**Figure 5:**
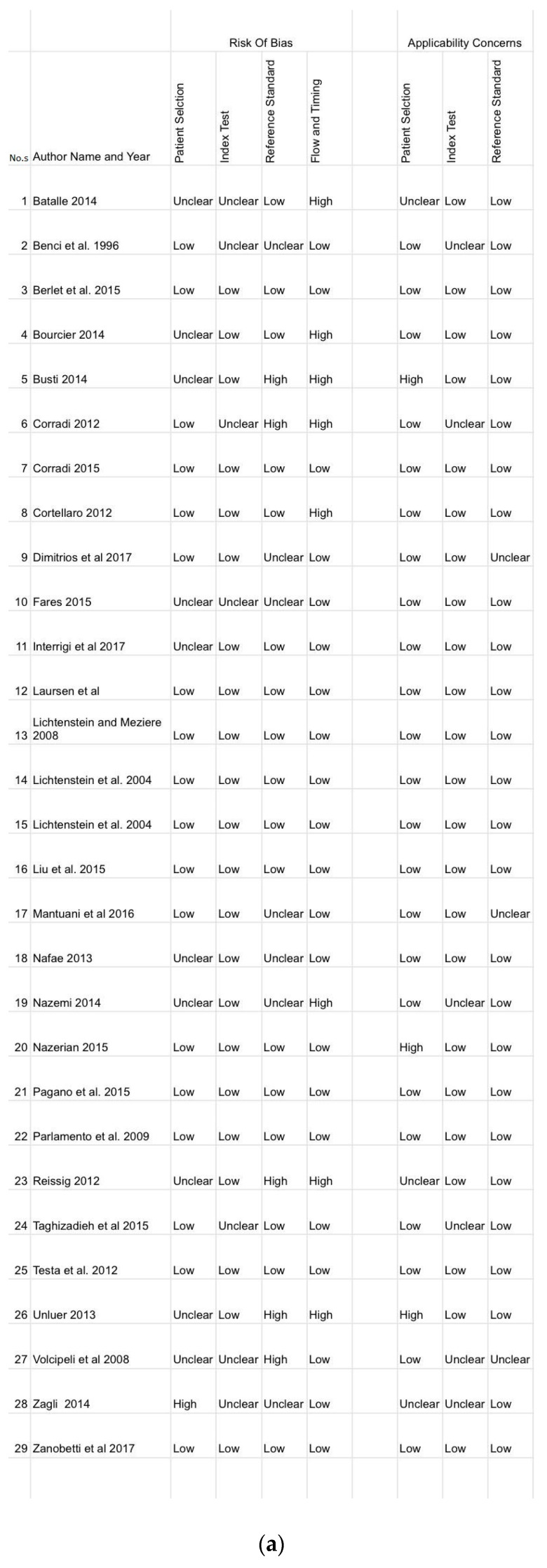

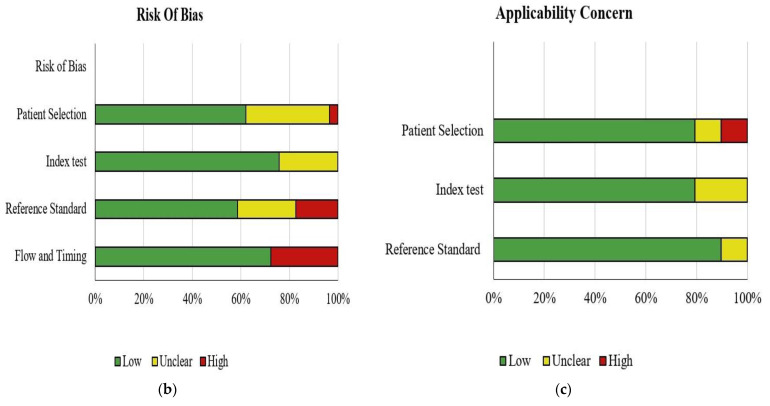
Risk of bias and applicability of concern of the 29 included studies. Image (**a**) shows the bias of every paper while image (**b**,**c**) show the frequency of a paper having a bias and their applicability concern respectively. These images show the quality of the papers used in the analysis.

The overall quality of studies included in our meta-analysis was fair (Figure 5). The publication bias in patient selection was low in 18, unclear in 10 and high in only one paper. However, the index test was low in 22 and unclear in 7. The appraised reference standard was low in 17, high in 5 and unclear in 7. The flow and timing were low in 21 and high in 8. Applicability concerns in patient selection was low in 23, high in 3 and unclear in 3. The index test was low in 23 papers and unclear in 6, while the reference standard was low and unclear in 26 and 3, respectively.

## 4. Discussion

LUS has only recently been appreciated by the wider medical community [57,58,59,60,61,62,63,64,65] because respected sources considered it to be unfit for assessing the pulmonary parenchyma [66] even though, during the past decade, LUS has been shown to be a very useful tool in the hands of intensivists and emergency physicians for the diagnosis of other thoracic conditions. Several studies have shown that bedside ultrasonography is useful for diagnosing cardiogenic pulmonary edema [67,68,69,70] is more accurate than CXR for diagnosing pneumothorax [71,72], and has applications in diagnosing pulmonary embolisms. Its use in the diagnosis of pneumonia has also been investigated in consideration of the great limitations of CXR. This is of particular importance when CXR is performed in the emergency departments, where many patients are critically ill and can be examined only in the supine position, often with bedside equipment [73].

Pneumonia commonly leads to significant pulmonary consolidation marked by a complete loss of aeration in the concerned lung region—manifesting differently in various modalities. On CXR, it is defined as a homogeneous opacity that may have effacement of blood vessel shadows and the presence of air bronchograms. Meanwhile, on LUS, consolidation is seen as an isoechoic, tissue-like pattern reminiscent of the liver, known as “hepatization”—with the aerated lung forming a boundary marked by the pleural line or an effusion if present. This potentially forms an irregular, scattered line if the consolidation is limited—specifically known as a “shred sign”—or a regular line if the whole pulmonary lobe is involved [5]. In addition, B-lines on LUS are well-defined hyperechoic comet-tail artifacts, arising from pleural lines and spreading vertically indefinitely, erasing A-lines and moving with the lung when lung sliding is present. It indicates the partial loss of lung aeration. However, consolidation is a non-specific sign of pneumonia because it is also present in lung atelectasis, and differential diagnosis could be difficult. The ultrasound sign that differentiates pneumonia from obstructive atelectasis is the presence of a dynamic air bronchogram in the former case (specificity 94% and positive predictive value 97) [32,63]. The possibility of a dynamic evaluation gives ultrasound an advantage over CXR, and possibly also over CT scan, which cannot always clearly differentiate between the two conditions [64].

Another distinct advantage of LUS in imaging pneumonia includes the better visualization of the regional pulmonary blood flow within lung consolidations in LUS with Doppler or contrast-enhanced sonography compared to CXR; thereby, providing critical information about the etiology of the disease. However, for all its benefits in detecting pneumonia in superficial lung parenchyma, LUS reliability remains doubtful in deeper alveolar lesions [57].

Our results suggest that bedside lung ultrasound has excellent accuracy for the diagnosis of pneumonia in adults. This points towards a clearly defined application of LUS as a diagnostic tool that can be considered reliable and dependable in clinical settings; further supplemented by a weighted sensitivity and specificity of 94% and 96%, respectively, with an area under the SROC curve of 0.98 according to Chavez et al. [11]. To further aid these findings, Hu et al. obtained a DOR of 509.99 and an area under the SROC curve of 0.99; although seven of the nine analyzed studies included children and even infants, so the samples were not comparable. In fact, several pediatric studies have suggested superior diagnostic performance for chest ultrasound in children compared to adults, which may be related to the fact that children usually have a thinner chest wall and a smaller volume of lung parenchyma, as outlined pertinently in the current literature [19,57,58]. When compared to other modalities currently considered for diagnosis, LUS was found to have various advantages such as (1) shorter turnaround time—with approximately 13 min being required in the procedure [59,60], along with (2) better reproducibility, (3) low cost, (4) avoidance of exposure to ionizing radiation, and (5) a broad spectrum of use in exploring findings not clearly visualized or understood on CXR [61,62]. This results in the determination of concrete evidence favoring a specific differential that would allow effective treatment regimens to be undertaken.

However, as also very prudently pointed out in the same studies, LUS also presents with a unique set of disadvantages such as (1) its limited value in patients with subcutaneous emphysema and in obese people due to the thickness of the chest wall; (2) inability to be conducted where access to the patient’s chest is limited by large bandages, prosthetic material or skin disorders; and most notably (3) its observer-dependent nature, as it implies the need for operators with certain skills and experience.

Although it has proven benefits, the inculcation of USG with different diagnostic techniques as a supplemental aid for reaching the correct diagnosis should be further studied, especially in the context of resource-limited settings [40]. One such study established that the addition of point-of-care ultrasonography (POCUS) of the heart, lungs and deep veins to the standard initial diagnostic tests resulted in 24% more patients with respiratory complaints being given correct presumptive diagnoses four hours after admission to the emergency department—yielding 21% more patients receiving appropriate treatment. However, the proportion of advanced diagnostic tests ordered was also higher in the POCUS group, possibly making it less cost-effective.

## 5. Conclusions

All aspects duly considered, ultrasound as a modality promises efficiency, efficacy and prudence in reaching an early diagnosis and can be safely employed for this purpose in patients suffering from pneumonia and a spectrum of cardiorespiratory conditions of varying etiologic and epidemiological factors.

## Figures and Tables

**Table 1:** Main characteristics of the included studies.

Study	Blinding	LUS Operator (Experience)	Reference Standard	Design	Country
Bataille, 2014 [1]	Unclear	Two researchers (NS)	Final diagnosis	Prospective	France
Benci, 1996 [35]	Yes	Experienced physicians	CD or CXR + CCT	Prospective	Italy
Berlet, 2015 [7]	Yes	Four Intensivists (NS)	Final diagnosis	Prospective	Switzerland
Bourcier, 2014 [8]	Yes	Five trained emergency physicians	Final diagnosis	Prospective	France
Busti, 2014 [12]	Yes	Expertise physician	CXR ± CCT	Prospective	Italy
Corradi, 2012 [36]	Unclear	NS	CXR ± CCT	Prospective	Italy
Corradi, 2015 [37]	No	NS	CCT	Prospective	Italy
Cortellaro, 2012 [15]	Yes	Emergency Physician (NS)	CXR/CCT	Prospective	Italy
Dimitrios, 2017 [38]	No	Emergency Physician (NS)	Final diagnosis	Prospective	USA
Fares, 2015 [16]	Yes	One physician (NS)	CCT	Cross-sectional	Egypt
Interrigi, 2017 [39]	No	Emergency Physician (NS)	CXR/CCT	Prospective	Italy
Laursen, 2014 [40]	Yes	Emergency Physician (>400 LUS)	Final diagnosis	Prospective	Denmark
Lichtenstein, 2008 [41]	Yes	Experienced physicians	CD or CXR ± CCT	Prospective	France
Lichtenstein, 2004 [42]	Yes	Experienced physicians	CCT	Prospective	France
Lichtenstein, 2004 [43]	Yes	Two ED physician sonographers	CCT	Prospective	France
Liu, 2014 [44]	Yes	Emergency Physician (28 h/50 LUS)	CCT	Prospective	China
Mantuani, 2016 [45]	Yes	Emergency Physician (NS)	Final diagnosis	Prospective	USA
Nafae, 2013 [46]	Yes	One physician (NS)	CCT	Cross-sectional	Egypt
Nazemi, 2014 [47]	Yes	Radiologist (NS)	Final diagnosis	Cross-sectional	Iran
Nazerian, 2015 [48]	Yes	Emergency Physician (>1 year)	CCT	Prospective	Italy
Pagano, 2015 [49]	Yes	Emergency Physician (NS)	Final diagnosis	Prospective	Italy
Parlamento, 2009 [50]	Yes	Emergency Physician (>10 years)	CXR/CCT	Prospective	Italy
Reissig, 2012 [30]	Yes	Experienced physicians	CXR ±CCT	Prospective	Germany
Taghizadieh, 2015 [51]	No	Emergency Physician (NS)	CXR/CCT	Prospective	Iran
Testa, 2012 [52]	Yes	Experienced physicians	CD or CXR ± CT	Prospective	Italy
Unluer, 2013 [53]	No	Emergency Physician (>6 h)	CXR/CCT	Prospective	Turkey
Volpicelli, 2008 [54]	Yes	Emergency Physician /radiologists (>200 LUS/year)	CXR	Prospective	Italy
Zagli, 2014 [55]	Unclear	NS	Final diagnosis	Case-control	Italy
Zanobetti, 2017 [56]	Yes	Emergency Physician (>80 h/150 LUS)	Final diagnosis	Prospective	Italy

Abbreviations: CCT, chest computed tomography; CXR, chest X-ray; CD, clinical diagnosis; ED, emergency department; LUS, lung ultrasound; NS, not specified.

**Table 2:** Main characteristics of the included participants.

Study	Sample Size	Mean Age (Years)	M/F	Setting	Inclusion Criteria	Diagnostic Criteria
Bataille, 2014 [1]	136	68	79/57	ICU	RF	Consolidation
Benci, 1996 [35]	57	38.5	unclear	Ward	Suspected CAP	Consolidation
Berlet, 2015 [7]	57	61.3	34/23	ICU	MV not for respiratory cause	Consolidation
Bourcier, 2014 [8]	144	78	72/72	ER	Suspected CAP	Consolidation or focal B lines
Busti, 2014 [12]	69	77.6	Unclear	Stroke Unit	Suspected CAP	Consolidation
Corradi, 2012 [36]	35	67.1	18/17	ER	Suspected CAP	Consolidation + focal B lines
Corradi, 2015 [37]	32	62	17/15	ER	Suspected CAP	Consolidation + focal B lines
Cortellaro, 2012 [15]	120	69	77/43	ER	Suspected CAP	Consolidation + focal B lines
Dimitrios, 2017 [38]	115	61	47/68	ER	Acute dyspnea	NS
Fares, 2015 [16]	38	61	Unclear	ICU	Suspected CAP	Consolidation
Interrigi, 2017 [39]	370	NS	NS	ER	Acute dyspnea	Consolidation
Laursen, 2014 [40]	158	73	61/97	ER	Acute dyspnea	Consolidation + focal B lines
Lichtenstein, 2008 [41]	260	68	140/120	ICU	Acute respiratory failure	Consolidation + focal B lines
Lichtenstein, 2004 [42]	32	58	Not mentioned	ICU	Acute respiratory distress syndrome	Consolidation
Lichtenstein, 2004 [43]	117	53	37/23	ICU	Chest pain or severe thoracic disease	Consolidation
Liu, 2014 [44]	179	72	100/79	ER	Suspected CAP	Consolidation + focal B lines
Mantuani, 2016 [45]	57	58	36/21	ER	Acute dyspnea	B lines
Nafae, 2013 [46]	100	Unclear	56/44	ICU	Suspected CAP	Consolidation
Nazem, 2014 [47]	151	61.44	Unclear	Ward	Suspected CAP	Consolidation
Nazerian, 2015 [48]	285	71	133/152	ER	Suspected CAP	Consolidation + focal B lines
Pagano, 2015 [49]	105	58	59/46	ER	Suspected CAP	Consolidation + focal B lines
Parlamento, 2009 [50]	49	61	31/18	ER	Suspected CAP	Consolidation + focal B lines
Reissig, 2012 [31]	362	64	228/134	ER and ward	Suspected CAP	Consolidation + focal B lines
Taghizadieh, 2015 [51]	30	NS	NS	ER	Suspected CAP	NS
Testa, 2012 [52]	67	55	Not mentioned	ER	Suspected H1N1 infection	Consolidation + focal B lines
Unluer, 2013 [53]	72	66	35/37	ER	Suspected CAP	NS
Volpicelli, 2008 [54]	217	67	132/85	ER	NS	Focal B lines
Zagli, 2014 [55]	221	56	152/69	ICU	Cases of VAP, controls without VAP	Consolidation
Zanobetti, 2017 [56]	2683	71	1367/1316	ER	Acute dyspnea	Consolidation + focal B lines

Abbreviations: CAP, community acquired pneumonia; ER, emergency room; ICU, intensive care unit; RF, respiratory failure; VAP, ventilator-associated pneumonia; NS, not specified.

## Data Availability

Data are available from the corresponding author upon request.

## Abbreviations

CAP, community-acquired pneumonia; CT, computerized tomography; CXR, chest X-ray; LUS, lung ultrasound; POCUS, point-of-care ultrasonography; ROC, receiver-operating-characteristic.
